# Why Aren't Antenatal Care Providers Adopting Oral Health Guidelines? A Qualitative Exploration

**DOI:** 10.1111/cdoe.13030

**Published:** 2025-02-18

**Authors:** Annika Wilson, Cailin Davies, Silvana Bettiol, Heather Bridgman, Leonard Crocombe, Ha Hoang

**Affiliations:** ^1^ Centre for Rural Health, College of Health and Medicine University of Tasmania Launceston Tasmania Australia; ^2^ Oral Health Services Tasmania Tasmanian Department of Health New Town Tasmania Australia; ^3^ Tasmanian School of Medicine, College of Health and Medicine University of Tasmania Hobart Tasmania Australia; ^4^ Violet Vines Marshman Centre for Rural Health Research La Trobe University Bendigo Victoria Australia

**Keywords:** oral health, prenatal care, professional practice gaps, qualitative research

## Abstract

**Objective:**

The current Australian Pregnancy Care guidelines recommend that antenatal care providers discuss oral health, provide advice and refer women to dental professionals as needed. However, the delivery of oral health recommendations in antenatal settings appears substandard. This study aimed to identify the barriers and enablers influencing antenatal care providers' adoption of the oral health guidelines.

**Methods:**

A qualitative study was conducted using semi‐structured interviews with a purposive sample of antenatal care providers in Tasmania, Australia. Data analysis followed a thematic coding method using the Capability, Opportunity, Motivation ‐ Behaviour (COM‐B) model and Theoretical Domains Framework to identify implementation barriers and enablers and to inform recommendations.

**Results:**

Twenty‐five antenatal care providers participated (midwives *n* = 14, general practitioners *n* = 10 and obstetrician‐gynaecologist *n* = 1). Thirty‐two explanatory themes were identified and mapped directly to six COM‐B constructs and 11 Theoretical Domains Framework domains. Eight main themes were identified as both impeding and enabling when viewed in different contexts: (1) perceived patient knowledge and awareness; (2) professional oral health knowledge, training and skills; (3) awareness of the guidelines on oral health; (4) patient education and professional resources; (5) interprofessional collaboration and support; (6) streamlined referral processes and access to dental services; (7) perceived outcomes of oral health interventions; and (8) perceived professional responsibility related to oral health.

**Conclusions:**

Key strategies include improvements to the promotion and dissemination of relevant guidelines, professional education and training, and development and adoption of oral health‐centred models of care to support interprofessional collaboration. Future research should focus on developing brief and sustainable interventions that address antenatal care providers’ practice behaviours.

## 1 Introduction

Oral health is an integral component of overall health, well‐being and quality of life [1]. Pregnant women are at increased risk of developing oral diseases such as dental caries, periodontal disease and pregnancy granulomas due to physiological, social and behavioural changes [2]. Periodontal disease has also been linked with adverse pregnancy outcomes such as preeclampsia and low birth‐weight deliveries, although the mechanisms are debated [3]. Studies show that vertical transmission of cariogenic microorganisms from mother to offspring increases susceptibility to dental caries and gingivitis in children [4]. Incorporating personal preventative measures for oral health, including maintaining good oral hygiene, reducing the consumption of sugary food and beverages, and seeking regular dental care, can significantly mitigate poor oral health outcomes. However, such measures can place substantial responsibility on women to not only initiate these activities but also understand the reasons why and how they contribute to oral health improvement [5]. Besides dental professionals, informed health professionals involved in antenatal care (ANC) can support women to improve their oral health by providing targeted education and behavioural change interventions.

Clinical practice guidelines remain a cornerstone of standardised care and inform health professionals to promote evidence‐based practice and enhance patient outcomes. The *Pregnancy Care* guidelines were first released in 2012 by the Australian Department of Health and have undergone several updates [6]. These evidence‐based guidelines include key oral health recommendations that advocate that all ANC providers assess pregnant women's oral health status, provide preventative advice and refer to dental professionals. However, contemporary systematic reviews have identified gaps in translating research into settings that integrate oral health and ANC [7, 8], which may explain why preventative oral health care is not routinely delivered by ANC providers. Studies have also identified multidimensional barriers to ANC providers adopting oral health care. These include a lack of oral health‐related knowledge and skills, limited resources, inadequate systems and tools, time constraints, perceived disinterest and attitudes that view oral health as outside their professional responsibility [8, 9].

Identifying factors that influence providers' practice behaviours remains a critical step in the process of translating evidence into practice [10, 11]. Understanding the role of ANC providers in oral health care is essential for developing interventions that effectively address these factors, align with clinical recommendations and are adapted to the specific needs and challenges of their healthcare environment. Recent guidance from the United Kingdom's Medical Research Council and the National Institute for Health Research proposes that iteratively developed multi‐component interventions are more likely to succeed when underpinned by behavioural theory as compared to those that lack a clear theoretical approach [12]. The Capability, Opportunity, Motivation‐Behaviour (COM‐B) model identifies behaviour constructs that can be modified to induce change [13], and is contingent on three assumptions: (i) the individual's ‘capability’ to enact the behaviour; (ii) the presence of ‘opportunity’ to facilitate the behaviour; and (iii) the ‘motivation’ to engage in the behaviour while overcoming conflicting motivations associated with competing behaviours. The Theoretical Domains Framework (TDF) comprises 14 domains that are mapped onto the COM‐B model, allowing for a more detailed analysis of the proximal determinants influencing behaviour [14]. The combined COM‐B and TDF model (herein identified as the COM‐B/TDF model) has been widely implemented across healthcare settings including stroke management in primary care [15], hand hygiene behaviours in hospitals [16] and simulated dental caries management in general dental practice [17].

The study aimed to answer the following research question: What barriers and enablers influence ANC providers' adoption of the oral health guidelines?

## 2 Methods

### 2.1 Study Design and Setting

A qualitative descriptive study design [18] was employed to explore the study aim. A qualitative approach was chosen as the most appropriate for this study as it enabled an in‐depth exploration of the complex factors influencing ANC providers' practice behaviours in adopting oral health guidelines [18, 19]. Data collected involved individual semi‐structured interviewing, with the choice for small focus group interviews [20]. The reporting of this study is in accordance with the Consolidated criteria for Reporting Qualitative studies (COREQ) 32‐item checklist [21]. Ethical approval was obtained from the Human Research Ethics Committee at the University of Tasmania (Reference: H0025029 and H0017221).

ANC providers were recruited in metropolitan, regional and rural areas of Tasmania, Australia, which is an Island state with an estimated population of 558 000 [22]. The capital city of Hobart is situated in the southern region of Tasmania, with most Tasmanians occupying inner‐regional towns, and smaller proportions of the population distributed across remote to very‐remote communities in the South, North and North‐West regions of the state [22]. Antenatal services are delivered in the private and public sectors under various models of maternity care, including: midwifery group practice, general practitioner (GP) shared care, private obstetrician and midwifery care, and public hospital maternity or high‐risk maternity care [23]. Recent 2022 data estimated 5498 registered births in Tasmania, where the median maternal and paternal age was 30.8 and 32.4 years, respectively [24].

### 2.2 Sample and Recruitment

Recruitment occurred between July and November 2021 and employed a maximum variation invitation strategy to ensure a diverse range of participant qualifications, ANC settings and geographic locations. However, the final sample reflected only those who responded to the invitation and did not fully achieve the intended maximum variation in participant characteristics. Providers were eligible to participate if they met the following criteria: (1) held clinical registration as an obstetrician or obstetrician‐gynaecologist, GP, midwife, nurse, Aboriginal and Torres Strait Islander (ATSI) healthcare worker or multicultural healthcare worker, as defined in the *Pregnancy Care* guidelines related to oral health [6]; (2) were 18 years of age or older; (3) residents of Tasmania, Australia; and (4) spoke English. The study was advertised by newsletters and invitations via post or email to relevant clinics, organisations and hospitals as listed within the Tasmanian Health Directory [25] across all regions. Telephone calls were made to individual practice managers by the first author (A.W.) to further promote the study. Participants were also able to recommend colleagues via a snowballing strategy. No participants were known to the research team prior to study participation.

### 2.3 Interview Schedule

A semi‐structured interview schedule was developed by the research team based on the study's aim, clinical recommendations (see Table 1) and review of the relevant literature. The schedule comprised 16 open‐ended questions related to participants' understanding of the role of oral health care during pregnancy, consequences of poor oral health, and the perceived barriers and enablers to adopting oral health guidelines. Participants' professional and demographic characteristics were also collected. The schedule was piloted with three ANC providers who were not included in the study, where feedback was documented and amendments made until consensus. The final interview schedule is presented in Data S1.

**TABLE 1:** Clinical oral health recommendations from the *Clinical Practice Guidelines: Pregnancy Care* [6].

When	At antenatal visits
Who	Midwives, GPs, obstetricians, ATSI healthcare workers, multicultural healthcare workers
What	**1. Discuss oral health with women:** Explain that pregnancy does not cause dental problems but may make them more likely. Advise women to have their oral health checked and to tell the dentist that they are pregnant
	**2. Provide advice on oral health to women experiencing nausea and vomiting:** Explain that vomiting exposes teeth to acid and give tips on how to reduce the impact
	**Practice summary:** At the first antenatal visit, advise women to have oral health checks and treatment, if required, as good oral health is important to a woman's health and treatment can be safely provided during pregnancy

### 2.4 Procedure

Twenty‐five providers contacted the research team by email and were provided with the Study Information Sheet and Consent Form after initial eligibility screening. All providers who approached the research team were eligible and agreed to participate in the study. Eligible participants elected for individual one‐on‐one semi‐structured interviews for approximately 30–35 min via video call (*n* = 4), telephone (*n* = 13) or face‐to‐face (*n* = 8). Written informed consent was obtained from each participant prior to interviewing. The interviews were audio‐recorded and conducted by a member of the research team with experience in qualitative research (A.W.). All participants were offered a $100.00 voucher to thank them for their participation.

Recruitment and data collection concluded upon reaching data saturation, indicated by conceptual saturation (where no new themes or patterns emerged) and theoretical saturation (where no additional insights further refined the theoretical framework) [26, 27]. Interviews were modified to remove identifying information and transcribed verbatim using an external organisation. Transcripts were uploaded to NVivo Version 12 (QSR International, Melbourne, Australia) qualitative analysis software. Transcripts were not returned to participants for comment or correction, and direct participant feedback on findings was not sought due to resource constraints.

### 2.5 Data Analysis

Interviews were analysed iteratively and recursively throughout the data collection phase. Analysis involved both inductive thematic analysis [28] and framework analysis [13] using the COM‐B/TDF model to identify barriers and enablers to implementing oral health recommendations by ANC providers. Inductive thematic analysis allowed flexibility for new insights to emerge, while the COM‐B/TDF model served as a structured deductive framework to ensure comprehensive coverage of all relevant behavioural determinants [13, 29]. These approaches were fully intertwined, with themes emerging from the data continuously mapped onto the COM‐B/TDF framework in an overlapping process. Further details on the application of the COM‐B model and TDF are provided in Data S2. Analysis was conducted by authors (A.W./C.D.) following Braun and Clarke's six‐step approach [28], with review and feedback from authors (S.B./H.B./L.C./H.H.) to ensure alignment with the study aims. Regular team meetings were held to discuss findings until consensus was reached.

### 2.6 Researcher Reflexivity

A.W. (MPH) conducted all qualitative interviews with participants. A.W. is a female PhD candidate with experience in qualitative research in dental public health and maternal health, which could have not only influenced interview discussions with additional prompts but also enhanced the understanding and interpretation of the context through insider knowledge [30]. Researchers involved in the analysis (A.W./C.D. [BDS, female]) are registered dentists, which could have assisted in bringing additional insight to the analysis of the data. The remaining authors S.B. (PhD, female), H.B. (DClinHlthPsych, female), L.C. (PhD, male) and H.H. (PhD, female) hold extensive research experience in maternal health, dentistry and behavioural change interventions. These authors regularly reviewed and provided feedback to ensure study rigour [31].

## 3 Results

A total of 25 ANC providers took part in the study and comprised midwives (*n* = 14), GPs (*n* = 10) and an obstetrician‐gynaecologist (*n* = 1). No ATSI or multicultural healthcare workers were recruited. Most participants were female (96%), aged between 18–39 (36%) and 40–59 years (44%), based in North‐West Tasmania (56%), with ≥ 16 years of clinical experience (60%). Demographic data are presented in Table 2.

**TABLE 2:** Demographic characteristics of study participants (*N* = 25).

ID	Tasmanian Region	MMM^a^	Qualification	Age group (years)	Gender	Highest academic attainment	Clinical experience (years)	Practice setting	Employment status	Pregnant patients seen per month
OB1	South	2	Obstetrician‐Gynaecologist	40–59	Female	Master's	≥ 16	Private Public	Full‐time	> 20
GP1	South	2	General Practitioner	18–39	Female	Graduate Certificate	11–15	Private	Part‐time	1–10
GP2	South	2	General Practitioner	40–59	Female	Bachelor's	≥ 16	Private	Part‐time	1–10
GP3	South	2	General Practitioner	18–39	Female	Bachelor's	6–10	Private	Part‐time	11–20
GP4	South	2	General Practitioner	40–59	Female	Master's	≥ 16	Private	Part‐time	1–10
GP5	South	2	General Practitioner	18–39	Female	Graduate Diploma	6–10	Private	Part‐time	> 20
GP6	South	2	General Practitioner	≥ 60	Female	Master's	≥ 16	Private	Part‐time	1–10
GP7	North	2	General Practitioner	18–39	Female	Master's	6–10	Private Public	Part‐time	11–20
GP8	North West	3	General Practitioner	40–59	Female	Graduate Diploma	11–15	Private	Full‐time	11–20
GP9	North West	6	General Practitioner	≥ 60	Male	Doctorate	≥ 16	Private	Part‐time	1–10
GP10	North West	3	General Practitioner	18–39	Female	Bachelor's	1–5	Public	Part‐time	1–10
MW1	South	5	Midwife	≥ 60	Female	Graduate Diploma	≥ 16	Private Public	Part‐time	1–10
MW2	South	2	Midwife	18–39	Female	Graduate Diploma	≥ 16	Public	Part‐time	1–10
MW3	North	2	Midwife	18–39	Female	Graduate Diploma	≥ 16	Private	Full‐time	> 20
MW4	North West	3	Midwife	40–59	Female	Graduate Diploma	≥ 16	Public	Part‐time	> 20
MW5	North West	3	Midwife	≥ 60	Female	Associate Diploma	≥ 16	Public	Part‐time	> 20
MW6	North West	3	Midwife	18–39	Female	Graduate Diploma	11–15	Public	Part‐time	> 20
MW7	North West	3	Midwife	40–59	Female	Bachelor's	≥ 16	Public Public	Part‐time	> 20
MW8	North West	3	Midwife	40–59	Female	Bachelor's	6–10	Public	Full‐time	> 20
MW9	North West	3	Midwife	40–59	Female	Associate Diploma	≥ 16	Public	Full‐time	> 20
MW10	North West	3	Midwife	≥ 60	Female	Graduate Diploma	≥ 16	Public	Part‐time	1–10
MW11	North West	3	Midwife	18–39	Female	Graduate Diploma	11–15	Public	Part‐time	> 20
MW12	North West	3	Midwife	40–59	Female	Bachelor's	≥ 16	Public	Part‐time	1–10
MW13	North West	3	Midwife	40–59	Female	Associate Diploma	≥ 16	Public	Full‐time	> 20
MW14	North West	3	Midwife	40–59	Female	Graduate Certificate	6–10	Private	Part‐time	11–20

^a^ Modified Monash Model [32]: a geographical classification model that categorises different areas in Australia into seven categories of remoteness (MM1 = metropolitan areas, MM2 = regional centres, MM3 = large rural towns, MM4 = medium rural towns, MM5 = small rural towns, MM6 = remote communities, MM7 = very remote communities) based on national Census data. The model provides guidance for the distribution of health care services, workforce and government‐funded programmes.

Participants identified barriers and enablers to implementing oral health recommendations, which were categorised according to their impact at the perceived patient, provider and service levels. Fifteen barriers were mapped to eight TDF domains and five COM‐B components, while 17 enablers were mapped to 11 TDF domains and six COM‐B components. Data S3 illustrates how participant quotes align with the COM‐B/TDF model.

### 3.1 Barriers

**FIGURE 1:**
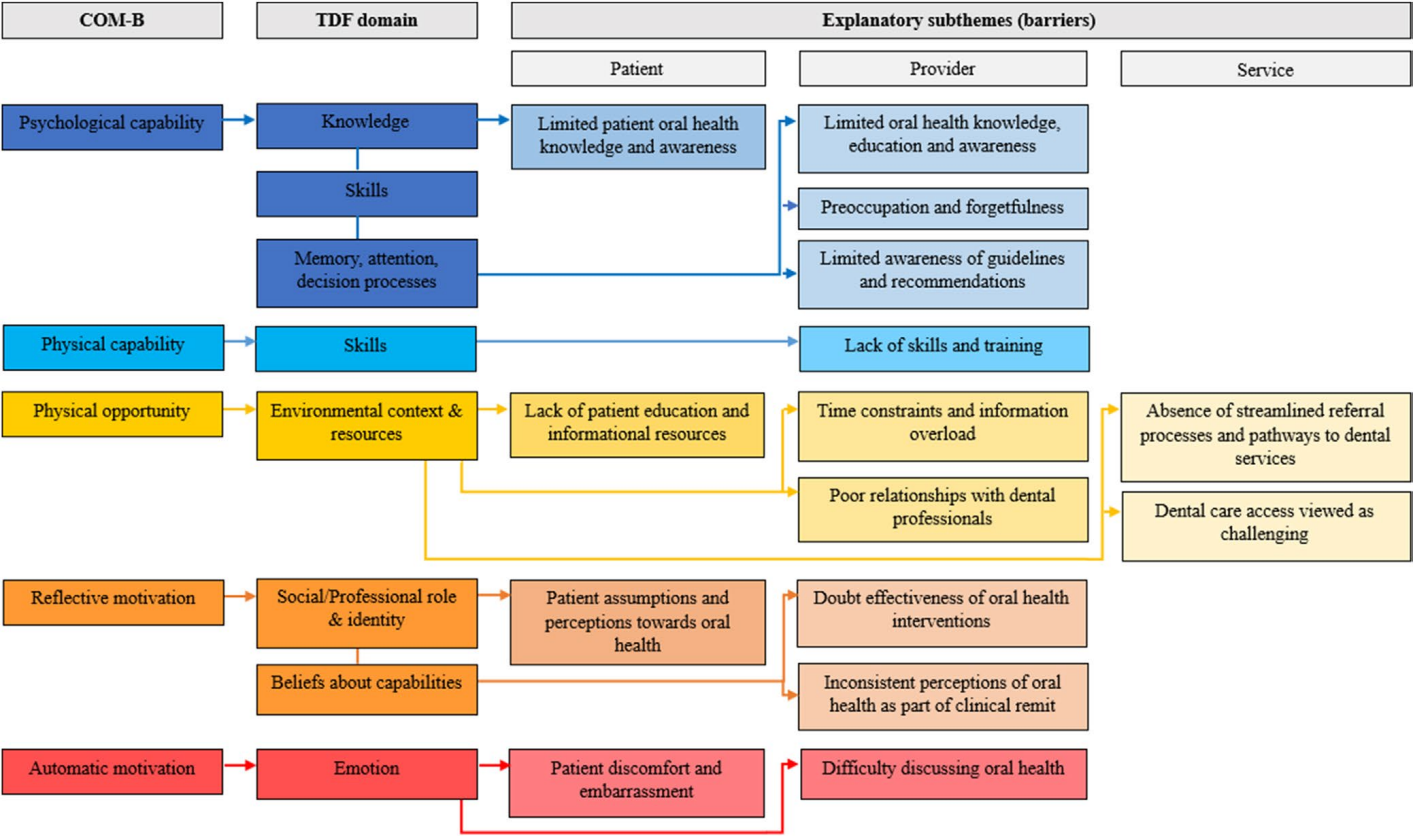
Mapping of explanatory subthemes (barriers) to the COM‐B/TDF model. This figure illustrates how the main COM‐B components link to specific TDF domains that influence ANC providers' practice behaviours in adopting oral health guidelines. Barriers are categorised into patient, provider and service levels to highlight distinct practice challenges associated with TDF domains (arrows). Certain barriers are linked to multiple TDF domains as they encompass different but related aspects of provider perceptions and attitudes that impact behaviour.

Numerous barriers emerged from the perspectives of ANC providers regarding oral health care. These barriers are described below and presented in Figure 1.

#### 3.1.1 Capabilities

Most providers expressed uncertainty regarding their *Psychological* and *Physical Capabilities* to implement preventative oral health care due to perceived deficiencies in their current knowledge, training and skills. Additional factors that impeded providers' *Psychological Capabilities* related to feelings of preoccupation with other clinical priorities, forgetfulness and limited to no familiarity of the oral health guidelines. Providers were also concerned about patients' limited knowledge and awareness of oral health, which discouraged them from initiating discussions due to a perceived lack of importance or necessity.

#### 3.1.2 Opportunities

Many providers were concerned about their *Physical Opportunities* to provide oral health care. *Physical Opportunities* were hampered by appointment time constraints, overburdening expectant parents with information and lack of patient education resources. Other barriers to providers' *Physical Opportunities* were uncertainty about referral pathways to dental services and the existence of organisational processes to support them. Many participants reported poor to non‐existent relationships with dental professionals in the community. Providers also noted challenges for patients in accessing dental care in Tasmania due to financial constraints (dental treatment was viewed as expensive) and long waiting lists, particularly in the public sector (thus, women viewed as unlikely to be seen during pregnancy).

#### 3.1.3 Motivations

Providers presented several negative beliefs that impacted their *Reflective* and *Automatic Motivations* to implement oral health care. Regarding their *Reflective Motivations*, providers were discouraged from discussing oral health due to their perceived ineffectiveness when delivered by them, and many providers felt that oral health fell outside of their clinical duties and responsibilities. Some providers held steadfast assumptions about pregnant women's disinterest in discussing oral health, unless it was perceived as directly relevant or needed. Regarding their *Automatic Motivations*, providers were fearful of provoking embarrassment or discomfort when addressing women's teeth due to their societal and aesthetic significance.

### 3.2 Enablers

**FIGURE 2:**
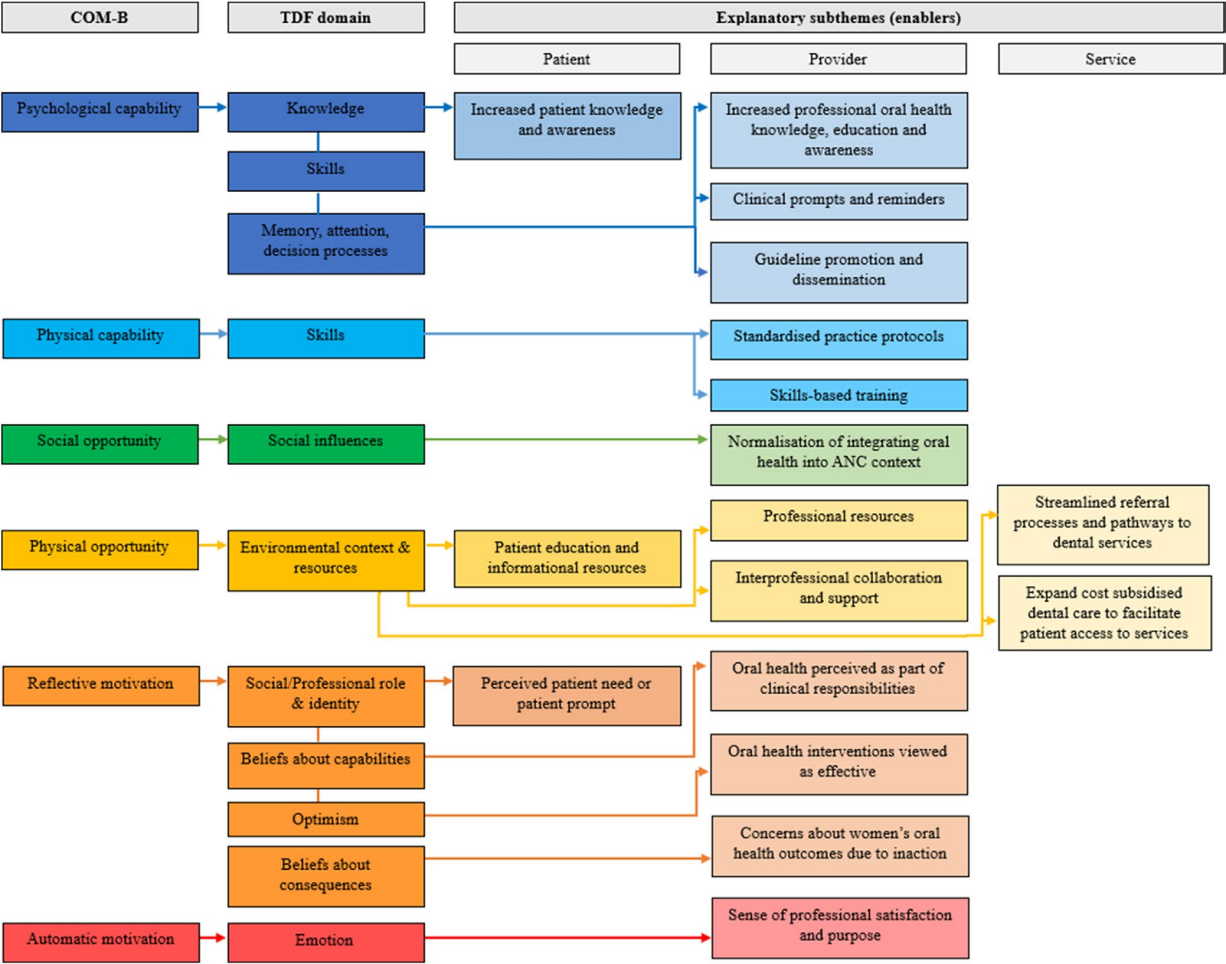
Mapping of explanatory subthemes (enablers) to the COM‐B/TDF model. This figure illustrates how the main COM‐B components link to specific TDF domains that influence ANC providers' practice behaviours in adopting oral health guidelines. Enablers are categorised into patient, provider and service levels to highlight distinct practice supports associated with TDF domains (arrows). Certain enablers are linked to multiple TDF domains as they encompass different but related aspects of provider perceptions and attitudes that impact behaviour.

Providers acknowledged several key enablers. Many of the barriers that providers initially reported were found to be enabling factors when viewed in different contexts. These are described below and in Figure 2.

#### 3.2.1 Capabilities

When it came to enhancing their *Psychological* and *Physical Capabilities*, most providers explained how improving their oral health knowledge, training and skills would lead to better implementation and adherence to recommendations. Examples of approaches to support their clinical practice included (1) promoting and disseminating the *Pregnancy Care* guidelines; (2) incorporating clinical prompts, reminders and organisational processes that include oral health components; and (3) undertaking skills‐based training and education. A few providers also reported an increased sense of *Psychological Capability* when they thought that patients were already aware and knowledgeable about their oral health.

#### 3.2.2 Opportunities

The development of professional and patient education and information resources was thought to facilitate providers' *Physical Opportunities* to implement recommendations. Brief and concise professional resources, like practical guidance posters, handouts and fact sheets, were desired in clinical rooms. While patient education and informational resources could include brochures, fact sheets, waiting room posters, or medical‐based apps. Many ANC providers highlighted a need to improve relationships with dental professionals to support their *Physical Opportunities* to provide oral health care. Streamlined referral processes within their workplace and expanding cost‐subsidised dental care were suggested to improve patient access to services and create more opportunities for referrals. Additionally, normalising the integration of oral health into ANC contexts could create *Social Opportunities* for providers to implement recommendations effectively.

#### 3.2.3 Motivations

ANC providers were highly motivated (*Reflective Motivations*) to engage in oral health care when they felt that interventions would be effective and improve the well‐being of their patients. Providers' *Reflective Motivations* were also influenced if they could easily see evidence of dental disease or if the patient expressed concerns about their own oral health. Many providers also felt compelled to address oral health issues when they realised how professional inaction could lead to unintended consequences such as worsening dental disease or adverse pregnancy outcomes. Regarding their *Automatic Motivations*, providers acknowledged that incorporating oral health care could offer professional satisfaction by helping women and addressing a broader public health issue.

## 4 Discussion

This is the first study to apply a theoretical framework to understand ANC providers' practice behaviours and highlights the challenges they face in providing oral health care. The results of this study identified critical sources of behaviour across all six constructs of the COM‐B model and 11 of the 14 TDF domains. Eight explanatory themes were identified as both impeding and enabling when viewed in different contexts: (1) perceived patient knowledge and awareness; (2) professional oral health knowledge, training and skills; (3) awareness of the *Pregnancy Care* guidelines related to oral health; (4) patient education and professional resources; (5) interprofessional collaboration and support; (6) streamlined referral processes and access to dental services; (7) perceived outcomes of oral health interventions; and (8) perceived professional responsibility related to oral health. Standalone barriers and enablers were also reported and included: (1) preoccupation and forgetfulness (barrier); (2) time constraints and information burden (barrier); (3) perceived discomfort and embarrassment when discussing oral health with patients (barrier); (4) concerns about women's oral health outcomes due to professional inaction (enabler); and (5) sense of professional satisfaction and purpose (enabler).

In general, ANC providers lacked the *Psychological* and *Physical Capabilities* in implementing oral health recommendations due to insufficient education and training. Recent systematic reviews have identified oral health knowledge and skills gaps among ANC providers [8, 33]. Given the time constraints reported by ANC providers as a barrier to oral health care [8, 34], it is recommended to deliver training using flexible approaches, preferably online [35], or in blended learning settings. Evidence of integrated learning approaches among ANC providers within Australia [36] and the United States [37, 38] has yielded positive professional outcomes including improved knowledge, confidence and competency. Furthermore, studies have demonstrated improvements to patient‐level outcomes because of oral health education and training among ANC providers. These outcomes included improved patient knowledge, positive clinical measures and increased dental service utilisation [39, 40, 41]. Improving the capabilities of providers could also strengthen their *Reflective* and *Automatic Motivations* by ensuring that their clinical interventions are effective, influential and aligned with contemporary evidence‐based practices. Strategies to enhance the promotion and dissemination of the *Pregnancy Care* guidelines on oral health [6] are also warranted, as many providers were unaware of the recommendations. Studies involving ANC providers have reported similar findings [9, 42]. Tailored awareness campaigns, implementation tools and guideline champions can effectively improve guideline uptake [43].

Both *Physical* and *Social Opportunities* were identified as crucial factors for supporting participants to implement oral health recommendations. ANC providers stressed the importance of interprofessional collaboration when promoting oral health to pregnant women. The emphasis on interprofessional collaboration reflects an understanding that no single healthcare discipline can adequately address the complex oral health challenges faced by pregnant women, and instead involves effective communication, shared responsibility and coordination among ANC providers and dental professionals. This aligns with similar evidence supporting the integration of oral health into pregnancy care by ANC providers [42], dental professionals [44] and pregnant women [45, 46]. Additionally, there is a need to create concise and easy‐to‐understand professional resources and patient education brochures to supplement practices. These resources could assist providers in conducting brief oral health assessments and providing education during appointments. Previous studies have demonstrated efficacy in the development of concise oral health guidance tools designed for ANC providers, enabling them to identify and assist women who are susceptible to poor oral health [34, 40]. Ensuring a linkage between these resources and existing dental referral pathways is crucial. Nevertheless, this presents a challenge in Australia due to the absence of dental care coverage under the publicly funded healthcare system, Medicare. Many ANC providers in this study also expressed that the high costs associated with dental care posed an additional barrier for patients seeking access to dental services. Efforts to bridge the gap between these two disciplines into existing healthcare models and systems could create opportunities for sustained interprofessional connections, strengthen existing referral pathways or develop new ones, and normalise the inclusion of oral healthcare into routine pregnancy care [47].

### 4.1 Limitations

Study limitations exist. First, the authors note the sampling bias given that participants comprised purposively selected Tasmanian ANC providers. Those who participated in the study may be more motivated towards oral health or feel inclined to provide socially desirable responses. Providers' motivations may be overestimated and not fully reflect the opinions of a wider antenatal population.

Second, purposive and snowball sampling may have introduced selection bias, particularly regarding the participation of public midwives in North‐West Tasmania. Despite efforts to recruit a diverse sample, no multicultural or ATSI healthcare workers participated. More women than men also participated in the qualitative study, which is consistent with literature suggesting greater male contribution in surveys [48]. However, there was a higher number of respondents from North‐West and South Tasmania, which may reflect a strength of the study in capturing rural and remote perspectives [48].

Third, the data used in the study, with the most recent data collected in 2021, may limit the applicability of the findings to the current context. Lastly, as this was a descriptive study, we elected not to compare provider types (e.g., midwives versus GPs) so as to prevent inaccurate or misleading results. Further research using diverse methods is needed to address these limitations, consider reasons for non‐participation and gather current perspectives from ANC providers of different types, genders, locations and service delivery models.

## 5 Conclusion

This study identified key barriers and enablers to adopting the *Pregnancy Care* guidelines on oral health by ANC providers. Significant challenges included gaps in professional knowledge, inadequate training and limited awareness of the guidelines, which could limit providers' ability to effectively address oral health concerns in pregnant women. Providers emphasised a need for brief, targeted interventions that offer clear, accessible clinical guidance and can be integrated into existing workflows and systems. Future research should prioritise the development of multi‐component interventions that address these practice barriers, improve guideline promotion and dissemination, strengthen professional education and training, and support the adoption of oral health–centred models of care.

## Author Contributions

Conceptualisation: Annika Wilson, Ha Hoang, Heather Bridgman, Leonard Crocombe and Silvana Bettiol. Methodology: Annika Wilson, Ha Hoang, Heather Bridgman, Leonard Crocombe and Silvana Bettiol. Software: Annika Wilson. Formal analysis: Annika Wilson, Cailin Davies. Investigation: Annika Wilson. Resources, Annika Wilson. Data curation: Annika Wilson. Writing – Original Draft Preparation: Annika Wilson. Writing – Reviewing and Editing: Annika Wilson, Cailin Davies, Ha Hoang, Heather Bridgman, Leonard Crocombe and Silvana Bettiol. Supervision: Ha Hoang, Heather Bridgman, Leonard Crocombe and Silvana Bettiol. All authors read and approved the final manuscript.

## Ethics Statement

Ethical approval was obtained from the University of Tasmania's Human Research Ethics Committee [Reference: H0025029 and H0017221].

## Consent

Informed written consent was obtained from all participants.

## Conflicts of Interest

The authors declare no conflicts of interest.

## Supporting information


Data S1.

Data S2.

Data S3.

## Data Availability

The data that support the findings of this study are available on request from the corresponding author. The data are not publicly available due to privacy or ethical restrictions.
