# Supplementary material for: Why Aren't Antenatal Care Providers Adopting Oral Health Guidelines? A Qualitative Exploration
Source: Community Dent Oral Epidemiol. 2025 Feb 18;53(3):286–95. doi: 10.1111/cdoe.13030 (PMC12064867; doi:10.1111/cdoe.13030)
Supplement: Supplementary file 1 — Data S1. [file CDOE-53-286-s003.docx]

**Data S1. Interview Schedule**

**Section 1. Interview questions.**

1. What does oral health mean to you?
2. What is your understanding of oral health during pregnancy?
3. If a pregnant woman asked you about oral health, what information would you tell her?
   - (Prompt) Can you give me an example of a time you discussed oral health with a pregnant woman? (Tell me about this).
   - (Prompt) When was the last time you discussed oral health with a pregnant woman?
   - (Prompt) How did you find this experience?
4. When would you decide to introduce the topic of oral health with pregnant women?

- (Prompt) Is there anything that would prompt you? (e.g., clinic guidelines, look of teeth, woman asks etc.)

1. Are you aware of the oral health guidelines for pregnancy?

- (Prompt) In your own words, what does the Clinical Guidelines recommend about oral health in pregnancy?
- (Prompt) When does the guideline recommend for you to advise a woman to have an oral health check or treatment?
- (Prompt) What does the oral health guideline recommend for women experiencing nausea and vomiting?

1. What do you believe is the purpose of the oral health guidelines for antenatal care?
2. What do you believe would happen if oral health wasn’t discussed as part of antenatal care?

Now I want to ask you some questions related to your confidence using a 0-10 scale, whereby 0 being not confident at all, and 10 being very confident.

1. So firstly, using this 0-10 scale, how confident are you discussing oral health with pregnant women?

- (Prompt) Can you expand on this? (Tell me more).

1. How confident are you in providing oral health advice for women experiencing nausea and vomiting?

- (Prompt) Can you expand on this? (Tell me more).

1. How confident are you referring a pregnant woman to a dentist?

- (Prompt) So you mentioned that you were least confident in *, can you elaborate why you feel that way?
- (Prompt) What could help you feel more confident?

1. What training have you had around oral health during pregnancy? (Tell me more).

- (Prompt) How prepared do you feel to provide oral health recommendations by this training?
- (Prompt) If you wanted to learn more about oral health during pregnancy, how would go about this?

1. Do you believe that oral health should be a component of your clinical practice? (Why/why not?)
2. What are some barriers that may make discussing oral health with women during pregnancy more challenging for you?
3. What are some enablers that may make this easier for you?
4. What specific systems or supports could help you implement the oral health guidelines?

- (Prompt) Are there any supports in your workplace/organisation to help you?

1. Is there anything else you would like to add that we haven’t yet touched on?

**Section 2. Demographic information.**

1. Qualification:
2. Primary work suburb(s):
3. Age Group
   1. 18-39
   2. 40-59
   3. ≥60
4. Gender:
   1. Male
   2. Female
   3. Prefer not to say
5. Years of experience in your clinical practice:
   1. <1
   2. 1-5
   3. 6-10
   4. 11-15
   5. ≥16
6. Type of setting:
   1. Private
   2. Public
   3. Other
7. Highest academic attainment:
   1. Associate diploma
   2. Bachelor’s
   3. Graduate Certificate
   4. Graduate Diploma
   5. Master’s
   6. Doctorate
8. Work type:
   1. Full-time
   2. Part-time
   3. Other
9. How many patients per month would you see that are pregnant?
   1. 0
   2. 1-10
   3. 10-20
   4. ≥21
10. And finally, are you aware of the *Healthy Smiles for Two* program^1^ run by the Oral Health Services Tasmania?
    1. Yes
    2. No.

1. Oral Health Services Tasmania. Healthy Smiles for Two: Pregnancy and Dental Health Tasmanian Government Department of Health, Oral Health Services Tasmania. Updated December 7 2021. Accessed April 29 2024. https://www.health.tas.gov.au/health-topics/dental-health/dental-health-programs-and-initiatives/healthy-smiles-two-pregnancy-and-dental-health
