# Supplementary material for: Why Aren't Antenatal Care Providers Adopting Oral Health Guidelines? A Qualitative Exploration
Source: Community Dent Oral Epidemiol. 2025 Feb 18;53(3):286–95. doi: 10.1111/cdoe.13030 (PMC12064867; doi:10.1111/cdoe.13030)
Supplement: Supplementary file 2 — Data S2. [file CDOE-53-286-s001.docx]

**Data S2. Understanding the COM-B model and TDF**

COM-B constructs

TDF domains

Physical: Skills (physical)

Psychological: Knowledge; Skills (cognitive and interpersonal); Behaviour regulation; Memory, attention and decision processes

Capability

**Behaviour**

Opportunity

Physical: Environmental context and resources

Social: Social influences

Motivation

Reflective: Intention; Goals; Social/professional role and identity; Beliefs about capabilities; Beliefs about consequences; Optimism

Automatic: Reinforcement; Emotion

**Fig.** The COM-B model components and corresponding mapping to the TDF domains.

**Abbreviations:** COM-B=Capability, Opportunity, Motivation – Behaviour; TDF=Theoretical Domains.

**Explanation:**

The COM-B model and TDF are interrelated frameworks designed to understand and facilitate behaviour change. The COM-B model is widely applied to contextualise individual-level change and the determinants of what must occur to achieve behavioural change and/or adopt new behaviours [1]. It posits that that changing behaviour relies on three components: Capability (both physical and psychological, representing an individual’s capacity to engage in change), Opportunity (the physical and social factors within the environment that support or hinder behaviour), and Motivation (both reflective and automatic processes driving the willingness to change) [1-3].

The TDF builds on the COM-B model by offering a structured approach to identify specific barriers and enablers across 14 domains that influence behaviour [4, 5]. These domains include knowledge; skills; memory, attention and decision processes; behaviour regulation; social influences; environmental context and resource; social/professional role and identity; beliefs about capabilities; optimism; intentions; goals; beliefs about consequences; reinforcement; and emotion [4, 6]. Combined, the TDF domains provide a comprehensive categorisation of the underlying factors that influence behaviour change and complement the broader structure of the COM-B model (see Fig). Definitions of behaviour change concepts as mapped to the COM-B model and TDF are provided in the following Table:

| **Data S2 Table.** COM-B model and TDF of behaviour change domains and definitions. | | | |
| --- | --- | --- | --- |
| COM-B |  | TDF domain | Definition (Cane et al. [4]) |
| Capability | Psychological | Knowledge | An awareness of the existence of something |
|  |  | Skills (cognitive and interpersonal) | An ability or proficiency acquired through practice |
|  |  | Memory, attention and decision processes | The ability to retain information, focus selectively on aspects of the environment and choose between two or more alternatives |
|  |  | Behavioural regulation | Anything aimed at managing or changing objectively observed or measured actions |
|  | Physical | Skills (physical) | An ability or proficiency acquired through practice |
| Opportunity | Social | Social influences | Those interpersonal processes that can cause individuals to change their thoughts, feelings or behaviours |
|  | Physical | Environmental context and resources | Any circumstance of a person’s situation or environment that discourages or encourages the development of skills and abilities, independence, social competence and adaptive behaviour |
| Motivation | Reflective | Social/professional role and identity | A coherent set of behaviours and displayed personal qualities of an individual in a social or work setting |
|  |  | Beliefs about capabilities | Acceptance of the truth, reality or validity about an ability, talent or facility that a person can put to constructive use |
|  |  | Optimism | The confidence that things will happen for the best or that desired goals will be attained |
|  |  | Intentions | A conscious decision to perform a behaviour or a resolve to act in a certain way |
|  |  | Goals | Mental representations of outcomes or end states that an individual wants to achieve |
|  |  | Beliefs about consequences | Acceptance of the truth, reality, or validity about outcomes of a behaviour in a given situation |
|  | Automatic | Reinforcement | Increasing the probability of a response by arranging a dependent relationship, or contingency, between the response and a given stimulus |
|  |  | Emotion | A complex reaction pattern, involving experiential, behavioural, and physiological elements, by which the individual attempts to deal with a personally significant matter or event |

**References**

1. Michie S, van Stralen MM, West R. The Behaviour Change Wheel: A new method for characterising and designing behaviour change interventions. Implement Sci. 2011;6(1):42.

2. Handley MA, Gorukanti A, Cattamanchi A. Strategies for implementing implementation science: a methodological overview. Emerg Med J. 2016;33(9):660-4.

3. Keyworth C, Epton T, Goldthorpe J, Calam R, Armitage CJ. Acceptability, reliability, and validity of a brief measure of capabilities, opportunities, and motivations ("COM-B"). Br J Health Psychol. 2020;25(3):474-501.

4. Cane J, O’Connor D, Michie S. Validation of the theoretical domains framework for use in behaviour change and implementation research. Implement Sci. 2012;7(1):37.

5. McLellan JM, O'Carroll RE, Cheyne H, Dombrowski SU. Investigating midwives' barriers and facilitators to multiple health promotion practice behaviours: a qualitative study using the theoretical domains framework. Implement Sci. 2019;14(1):64.

6. Atkins L, Francis J, Islam R, O’Connor D, Patey A, Ivers N, et al. A guide to using the Theoretical Domains Framework of behaviour change to investigate implementation problems. Implement Sci. 2017;12(1):77.
