# Supplementary material for: Why Aren't Antenatal Care Providers Adopting Oral Health Guidelines? A Qualitative Exploration
Source: Community Dent Oral Epidemiol. 2025 Feb 18;53(3):286–95. doi: 10.1111/cdoe.13030 (PMC12064867; doi:10.1111/cdoe.13030)
Supplement: Supplementary file 3 — Data S3. [file CDOE-53-286-s002.docx]

**Data S3. Themes and illustrative quotes**

| **Data S3. Table 1.** Barriers to adopting oral health guidelines in ANC: Insights from COM-B/TDF analysis with illustrative quotes. | | | | |
| --- | --- | --- | --- | --- |
| **COM-B model** | **TDF domains** | **Explanatory barriers** | **Illustrative quotes** | **Description of theme** |
| **Psychological capability** | Knowledge | **Patient level:**  Limited patient oral health knowledge and awareness | ‘A lot of people don’t go to the dentist, and probably, if we didn’t mention it, they wouldn’t. A lot certainly aren’t aware of the acid and the brushing of your teeth afterwards.’ (GP9) | This theme highlights a significant gap in perceived patient awareness regarding the importance of oral health during pregnancy based on providers’ experiences. Patients are often unfamiliar with the risks associated with poor oral health or are unaware of safe dental practices while pregnant, indicating a potential lack of understanding in this area. |
|  |  |  | ‘I think a lot of ladies wouldn’t get the check up they needed. They’d think it would be not appropriate to have dental treatment during pregnancy. Because part of it is reassuring them any treatment is safe, otherwise we wouldn’t recommend it. But women don’t bring it up, so it’s not something I commonly do.’ (MW13) |  |
|  | Knowledge; Skills; Memory, attention and decision processes | **Provider level:**  Limited provider oral health knowledge, education and awareness | ‘…our awareness is really low, and it’s something that, actually, I think rarely gets discussed, to be honest with you. And there could possibly be poor outcomes for the mother and baby, I’m not sure if there’s a link with preterm labour and poor oral health. But yes, so once again, very, very poor awareness from the GP side.’ (GP3) | Providers commonly report limited knowledge and training related to oral health, especially within the context of pregnancy. This lack of awareness among providers leads to low confidence in discussing oral health, often resulting in it being overlooked during ANC. |
|  |  |  | ‘…coming from a place where I’ve got minimal oral health education, I guess I don’t feel well placed to discuss it at this stage as well.’ (MW14) |  |
|  |  | **Provider level:**  Preoccupation and forgetfulness | ‘I suppose it’s something that I probably need to put some more thought into… To be honest with you, it’s not something really that I had much of a thought of before.’ (GP1) | Providers indicated that oral health is not consistently front of mind in ANC, often due to preoccupation with other clinical tasks. This preoccupation suggests that oral health may not be perceived as an urgent issue, making it less likely to be remembered or discussed. |
|  |  |  | ‘Look, I think probably I have had some training. It’s probably been tackled in services during my time as a midwife, but I haven’t got a clear memory of that… There was probably some teaching around things in that but I don’t remember it at all.’ (MW1) |  |
|  |  | **Provider level:**  Limited awareness of guidelines and recommendations | ‘I have to admit I had never heard of oral health guidelines in pregnancy.’ (GP8) | There is a widespread lack of awareness among providers about specific oral health guidelines for pregnancy. This gap results in inconsistent application of oral health recommendations and contributes to uncertainty about what advice to offer. |
|  |  |  | ‘I’ve no idea what the oral health guidelines are for pregnancy, specifically. I assume that it would be something about getting your teeth checked and making sure everything is okay. But other than that, I don’t know.’ (GP6) |  |
| **Physical capability** | Skills | **Provider level:**  Lack of skills and training | ‘I don’t think I’ve ever had any training around oral health full stop, probably had one lecture in medical school… I’ve never had it… I’ve got an Advanced Diploma of Obstetrics and Gynaecology, and I’m an ongoing diplomat of the college, and I don’t think I’ve ever had any training.’ (GP2) | Many providers recall little to no formal training on oral health in their professional education, leaving them with limited skills in addressing these needs during ANC. This lack of training impacts their comfort and effectiveness in discussing or assessing oral health. |
|  |  |  | ‘I don’t know. I don’t I don’t actually remember learning much about it through my training at all. I think my… I have no recollection of doing anything around oral health in pregnancy as a medical student, as a resident or as a registrar. And I don’t actually ever recall seeing someone talk about oral health… at a conference for obstetrics either. But I think it’s a topic that in my experience has not been regularly covered.’ (OB1) |  |
|  |  |  | ‘It’s pretty much what you learn as you go along, really. I probably have never done a formal oral health course.’ (MW7) |  |
| **Physical opportunity** | Environmental context and resources | **Patient level:**  Lack of patient education and informational resources | ‘Probably Google it or look at RANZCOG if they had anything about it. I’m not really sure where else would have the information. I don’t know if RACGP has any information. I wouldn’t even know what the peak oral or dental health body is in Australia. I wouldn’t even know, to be honest. I’d probably be Googling something like Australian Dental Association and hoping that got me there, but I actually wouldn’t know.’ (GP2) | Providers perceive a general lack of accessible informational resources, meaning patients have limited means to obtain reliable guidance on oral health during pregnancy. This lack of materials constrains both patients’ understanding and providers’ ability to effectively educate them. |
|  |  |  | ‘In my I practice doesn’t hold any paraphernalia around oral health. I guess for people who are looking for more information, I don’t have a hardcopy resource to give them.’ (OB1) |  |
|  |  | **Provider level:**  Time constraints and information overload | ‘I suppose time would be the most critical barrier, because there is a lot to go through in antenatal appointments, and I do a lot of antenatal care. So, getting through results and tests needed, and examinations, blood pressure, all of that stuff, and checking on their social welfare, psychological welfare, all that kind of thing is time-consuming. And so, adding in another factor on top of that is… it is one of those things that you don’t put in the top priority list.’ (GP7) | Providers report that the time available in antenatal appointments is often insufficient to address oral health, especially given the extensive range of topics that must be covered. This time limitation leads oral health to be deprioritised relative to other, more immediate pregnancy-related concerns. |
|  |  |  | ‘The sheer volume of advice that we’re now expected to communicate to women in early pregnancy is not compatible with 15-minute medical appointments. It’s impossible. So, unless you’ve got a woman who is able to come back for two or three visits, or book a long appointment, it’s really challenging to do that.’ (GP2) |  |
|  |  | **Provider level:**  Poor relationships with dental professionals | ‘I can’t say I have any relationship with them at all.’ (GP8) | A lack of established relationships between ANC providers and dental professionals creates a barrier to coordinated care. This disconnect reduces the likelihood of referrals and limits interprofessional support for patients with oral health needs during pregnancy. |
|  |  |  | ‘Not concerning pregnancy. I’ll sometimes reach out for other medical issues, like cancer patients or high-risk patients. I will shamefully admit we don’t communicate as much as perhaps we should.’ (GP10) |  |
|  |  |  | ‘No. There isn’t really any relationship or communication with them about pregnancy… maybe the occasional email from them requesting some medical information. Otherwise, that’s it.’ (MW6) |  |
|  |  | **Service level:**  Absence of streamlined referral processes and pathways to dental services | ‘Private dentists, very few people can afford those. I wouldn’t know which private dentists are the cheapest. If someone said, look, I can go to a private dentist but tell me which ones are not too expensive, then I would have no idea about that.’ (GP6) | Providers experience challenges in navigating the referral process for affordable dental services, with no clear or standardised pathways. This lack of streamlined processes adds complexity to referrals and may discourage providers from addressing oral health needs. |
|  |  |  | ‘I’m not really aware of a clear process of referring women in my practice privately, other than to the public system, but even then I’m not too confident.’ (MW9) |  |
|  |  | **Service level:**  Dental care access viewed as challenging | ‘Money is the big one. You can give them generalised advice about what to do, but if they’ve got established dental caries, or established problems, then access to dental services is very difficult for them.’ (GP9) | Financial and logistical barriers significantly hinder access to dental care for many pregnant patients, particularly those from lower socioeconomic backgrounds. This challenge reflects the broader issue of limited access to affordable dental care within the community. |
|  |  |  | ‘…when I was working at the hospital, working with a cross-section of women, some migrants, some people from the poorer suburbs, they probably won’t even think of it, I would say. The cost of dentistry is quite difficult for a lot of people and not high on the priority list… You can go on a list here in Hobart to get some free dental checks through the public system, but you often wait six or nine months to do it, so that isn’t very helpful in pregnancy.’ (MW1) |  |
| **Reflective motivation** | Social/Professional role and identity | **Patient level:**  Patient assumptions and perceptions towards oral health | ‘Well, usually the most pressing issue is looking after the pregnancy, and so it seems, often, to women that oral health is a superfluous topic. And so there’s not a lot of attention, because they’re primarily focused on the health of the baby, and their health, especially if there is a lot of hyperemesis and nausea. So, the last thing on their minds is brushing their teeth and looking after things like that.’ (GP7) | Providers perceive that many pregnant patients view oral health as secondary to the immediate concerns of pregnancy and may not prioritise dental visits. This perception results in lower engagement with oral health advice and services during pregnancy. |
|  |  |  | ‘Probably the only challenging thing is those ladies that haven’t been to the dentist for a really long time, and even though you discuss the benefits, they sort of don’t see the need, I guess, to go to the dentist.’ (MW9) |  |
|  | Social/Professional role and identity; Beliefs about capabilities | **Provider level:**  Doubt effectiveness of  oral health  interventions | ‘…basically, oral health is actually really important for general health and general health outcomes and it’s not something that we often address, I think, because we’re not… Well, I feel I don’t because I’m not sure necessarily about the management and how effective it would be coming from us.’ (MW10) | Providers express uncertainty about the impact of oral health interventions within ANC, often questioning whether their advice or actions can meaningfully influence patient outcomes. This doubt affects their motivation to address oral health with patients. |
|  |  |  | ‘…I really don’t think that beside me bringing it up, I don’t think it would really change things.’ (GP10) |  |
|  |  | **Provider level:**  Inconsistent perceptions of oral health as part of clinical remit | ‘I think, in general, medical practitioners, the dental stuff is the one bit of medicine they say that we don’t need to know about. So, I think we almost joyfully embrace that. That’s the part that we don’t need to know about. So, we’re like, great, go and see someone else about that because everything else I have to know about. So, I think there’s a little bit of a, it’s not my area.’ (GP2) | ANC providers often see oral health as outside their professional scope, viewing it as the responsibility of dental professionals. This perception leads to a reluctance to engage with oral health issues and may result in missed opportunities to address patient needs within ANC settings. |
|  |  |  | ‘I’m not a dentist, so yes, would I like to know everything and be able to tell everything to everybody? I would love to, but do I know all the answers? No, because I’m not a dentist. But if I can pick up that there’s a problem and then refer on, then they’re going to get the right help that they need for their dental care.’ (MW4) |  |
| **Automatic motivation** | Emotion | **Patient level:**  Patient discomfort and embarrassment | ‘I guess it’s when you get those people that have, like, the really decayed teeth and things like that. That sometimes it’s embarrassing for them to talk about it, which makes it then a little bit embarrassing for you to talk about it. People are self-conscious about it and stuff like that, so sometimes that can be a barrier.’ (MW6) | Patients’ perceived embarrassment or discomfort in discussing oral health, particularly in cases of visible decay, creates a social barrier. This discomfort can affect the patient-provider dynamic, making oral health discussions less likely. |
|  |  | **Provider level:**  Difficulty discussing oral health | ‘I do find it difficult to bring up sometimes. Especially when I’m concerned about upsetting them or making them feel judged for something they can’t always control.’ (MW4) | Providers may feel hesitant to discuss oral health out of concern for patient sensitivities, especially if patients appear self-conscious about their dental issues. This emotional barrier can contribute to oral health being avoided or only briefly mentioned in consultations. |

Abbreviations: ANC=Antenatal care; GP=General Practitioner; RACGP=Royal Australian College of General Practitioners; RANZCOG=Royal Australian and New Zealand College of Obstetricians and Gynaecologists.

| **Data S3. Table 2.** Enablers to adopting oral health guidelines in ANC: Insights from COM-B/TDF analysis with illustrative quotes. | | | | |
| --- | --- | --- | --- | --- |
| **COM-B model** | **TDF domains** | **Explanatory enablers** | **Illustrative quotes** | **Description of theme** |
| **Psychological capability** | Knowledge | **Patient level:**  Increased patient oral health knowledge and awareness | ‘I think arming the patient with the right information so they know more about why we are talking about dental health would be really useful.’ (MW2) | This theme reflects a growing awareness and recognition among patients of the importance of oral health, especially when providers make an effort to educate them. Patients are more likely to understand and prioritise oral health when they receive clear, accessible information. |
|  |  |  | ‘Definitely making the patient more aware too by putting out more messages in the community, so when they come to an appointment and we start talking about dental care, they can go, oh yeah, that’s why.’ (GP8) |  |
|  | Knowledge; Skills; Memory, attention and decision processes | **Provider level:**  Increased provider oral health knowledge, education and awareness | ‘Probably more education towards that, because it’s actually something that we don’t really cover in depth.’ (MW7) | Providers demonstrate a willingness to increase their knowledge about oral health in pregnancy, acknowledging that enhanced education would enable them to better support patients. This reflects a proactive attitude among providers toward improving their own competency. |
|  |  |  | ‘I think it would be useful to have better education, particularly by dentists who can feed back what they’re seeing amongst pregnant populations.’ (GP7) |  |
|  |  |  | ‘More education. I can’t stress that enough.’ (GP1) |  |
|  |  | **Provider level:**  Clinical prompts and reminders | ‘The education’s usually done at booking in because we have an education checklist that we have certain times through the pregnancy.’ (MW14) | The use of clinical prompts and checklists supports providers in consistently addressing oral health with patients, ensuring it is integrated into routine care. Such reminders serve as useful cognitive aids for providers amid busy clinical environments. |
|  |  |  | ‘I think if I had a reminder to. I think it’s the kind of thing that would be very appropriate for patient handouts or checklists, so that we can run through a checklist, and if we don’t get to it necessarily in consult time, it can be flagged to come back to or something the patient can take away and consider because there’s just a vast tract of information to get through.’ (GP2) |  |
|  |  | **Provider level:**  Guideline promotion and dissemination | ‘…probably one of the best ways might be an update article within the General Practice magazine. So in our Australian Journal of General Practice, having an article in there that updates for GPs and points them to the guidelines, and gives them some advice.’ (GP8) | Providers express that regular guideline dissemination and updates help maintain awareness and adherence to recommended practices. When guidelines are readily accessible, providers are more likely to integrate them into patient care. |
|  |  |  | ‘I guess education, awareness of guidelines. It’s part of a tick-box, and part of our antenatal care discussion, or consult… Generally, we get guidelines sent out to us just via our email system, or message system. We get lots of guidelines sent out to us, so that might be one option, just to raise awareness amongst GPs.’ (GP4) |  |
| **Physical capability** | Skills | **Provider level:**  Standardised practice protocols | ‘I’m thinking the system we’ve got here in place at the moment. I think it’s working well, because we introduced it at booking in, to see if they have been to the dentist, and whether they’d like a referral to oral health. And then at 20 weeks we revisit it, and follow up on it, to see whether they’ve forgotten about it, or they’ve been contacted by Oral Health Tasmania, and follow it up that way. But that’s where we leave it. We make that initial one, we follow it up there… That’s our process here, and it helps us to make sure we cover it.’ (MW13) | Standardised protocols for discussing oral health in pregnancy promote consistent practice, allowing providers to systematically address oral health at specific points during ANC. This structured approach reinforces oral health as an integral part of care. |
|  |  | **Provider level:**  Skills-based training | ‘CPD opportunities. I’m constantly updating my knowledge, but I haven’t seen a lot about oral health in pregnancy out there that’s come without me actively seeking it. (GP7) | Providers recognise the value of skills-based training to enhance their ability to address oral health. This indicates a positive attitude toward learning and suggests that providers are open to further professional development in this area. |
|  |  |  | ‘Probably in-service maybe because it’s been a while… Yes, as a training for us, because we’re the ones that are talking to a majority of women before we refer them on.’ (MW4) |  |
| **Social opportunity** | Social influences | **Provider level:**  Normalisation of integrating oral health into ANC context | ‘Well, probably, that’s part of the normal process during pregnancy, is that we discuss this because it does have an impact. So, we discuss this with every woman during her pregnancy that this is part of her general health. And that that can impact on her pregnancy, so we want to make sure that that’s all looked after.’ (MW12) | There is a sense among providers that oral health discussions should be a normalised and routine part of ANC. By viewing it as part of general health, providers are more inclined to include oral health as a standard discussion topic. |
|  |  |  | ‘I think making it more accepted – more a part of our regular practice would encourage a lot of us to incorporate oral health into antenatal appointments, that’s for sure.’ (GP2) |  |
| **Physical opportunity** | Environmental context and resources | **Patient level:**  Patient education and informational resources | ‘We don’t have a list of dentists that are particularly interested in women who are pregnant, which might just be a subgroup of dentists. So that could be another resource that we could also have on hand to give to the patient.’ (OB1) | Providers see value in having specific informational resources that could be easily shared with patients, allowing them to independently access reliable oral health information. Resources tailored for pregnancy can make oral health education more relevant for patients. |
|  |  |  | ‘If I had a little section there about oral health, then I think that would be really useful, just to add in a sentence or two about it, just within that two-page PDF that you can print out for patients.’ (GP1) |  |
|  |  | **Provider level:**  Professional resources | ‘We all learn differently. So having another format of learning other than just verbal information. I think as a result, if you’ve got a written documents in or an oral intent-based document to then get further expansion on the information you get given.’ (OB1) | Providers appreciate the availability of varied, accessible resources to support their own learning. These professional resources allow them to quickly reinforce key points and better communicate oral health information to patients within limited timeframes. |
|  |  |  | ‘I guess if I had some easily accessible bite-sized bits of information to tell me the key points, and perhaps some ready resources to give. Understandably, getting a GP’s attention in what is a very crowded marketplace for information with multiple guidelines on multiple conditions to digest every day.’(GP2) |  |
|  |  | **Provider level:**  Interprofessional collaboration and support | ‘I talk to my own dentist sometimes. And he seems happy to chat. And yes, we chat amongst ourselves as well. Some of the dentists are great when it comes to hyperemesis in pregnancy, when they’ve suffered the same. They always seem willing to talk when I explain that I’m interested in referring so and so.’ (MW4) | Collaboration between ANC providers and dental professionals is viewed positively, as it facilitates shared knowledge and a more integrated approach to patient care. Such interactions allow providers to access specialised insights from dental professionals. |
|  |  |  | ‘Increasing my communications with local dentists would be good. They could give resources like information pamphlets that I could then pass on to the patients.’ (MW6) |  |
|  |  | **Service level:**  Streamlined referral processes and pathways to dental services | ‘I have an autofill for antenatals and referrals. So, if they’ve got a Health Care Card, I’ll give them that brochure for the, I want to say it’s Healthy Smiles, or something like the government, the public oral health program. It’s an automatic process.’ (GP5) | Streamlined referral pathways facilitate efficient patient navigation to dental services. These processes reduce the complexity of referring patients, making it easier for providers to connect patients with appropriate care. |
|  |  |  | ‘Definitely a process that points out how to refer women to a private dentist or to the public program would be immensely useful. We have to be aware of so many things, so something to lighten that mental load would be great. Perhaps something we could establish in the admin side or a form to fill out and give to women.’ (MW1) |  |
|  |  | **Service level:**  Expand cost subsidised dental care to facilitate patient access to services | ‘I believe it should be covered by Medicare, that’s the main thing. The reason it’s not part of our clinical practice, where I work, is because people can’t afford to see dentists. If we could refer people, if people could access dental care better, we would probably be more proactive about looking for issues and referring people.’ (GP6) | Providers recognise the importance of affordable dental care for pregnant women, acknowledging that financial barriers often hinder patient access. Subsidised dental care is seen as an enabler for broader patient engagement with oral health services. |
|  |  |  | ‘Oh, yes. It’s a public health measure. The public health unit in Tasmania should be out there holding up the banner for oral health, which includes getting dental checks, cleaning your teeth, dental hygiene, seeing your dentist or your doctor if you’ve got infected teeth or gums.’ They need to expand their reach to people and make it easier to access – make it affordable for people to get the care they need.’ (GP9) |  |
| **Reflective motivation** | Social/Professional role and identity | **Patient level:**  Perceived patient need or patient prompt | ‘…it’s just something we skim over, like all the other questions, unless the woman actually stops and spends time on it herself.’ (MW3) | Providers are more likely to engage with oral health if they perceive an explicit need or if prompted by the patient. This theme highlights that patient-initiated discussions or visible oral health issues can encourage providers to address dental care more thoroughly. |
|  |  |  | ‘Not routinely, but if I notice they’ve got bad dental caries or bad gum disease I do discuss it. I particularly focus on young mothers with kids, when they bring them in, if I note any dental issues with their children. When I’m just talking to the kids, I actually raise it with the mum whether the mum’s there as the patient, or the kid’s there.’ (GP9) |  |
|  | Social/Professional role and identity; Beliefs about capabilities; Optimism | **Provider level:**  Oral health interventions viewed as effective | ‘As long as I know that the recommendations would be useful and help patients, then yes, I would feel more comfortable to spend some time talking about dental health. I do understand that it’s important and affects the whole body.’ (MW2) | Providers who view oral health interventions as effective and impactful are more motivated to discuss them. This belief in the value of oral health care fosters a proactive approach to integrating these interventions into ANC. |
|  |  |  | ‘Again, you want future generations, so if you start pregnant, hopefully the future generations, it’s going to be yes, we need to go to the dentist, it’s good for our health. But you, as their guide, are also improving that mum’s health through that pregnancy as well, and long-term. And yes, it might be a pain, and it might be expensive if you don’t have a Healthcare card, but benefits for your long-term health are important. We know it works.’ (MW4) |  |
|  | Social/Professional role and identity; Beliefs about capabilities | **Provider level:**  Oral health perceived as part of clinical responsibilities | ‘For the prevention of untoward circumstances from poor oral health as a primary healthcare initiative. While we’ve got women here, it’s really great to educate on things that they may not have known about or been exposed to before. For their own benefit, for the benefit of their babies and of their children and their families, yes, absolutely.’ (MW11) | Many providers perceive oral health as a natural extension of their clinical role in pregnancy care and recognise its relevance to overall maternal and foetal health. This perception reinforces the integration of oral health into routine ANC practices. |
|  |  |  | ‘Yes, it’s part and parcel of pregnancy care, along with diet, physio, and all the other parts. It makes up the whole, because everything’s interrelated and has an impact.’ (MW13) |  |
|  | Beliefs about consequences | **Provider level:**  Concerns about women’s oral health outcomes due to inaction | ‘I think a lot of people would have really poor dental hygiene, and there’d be tooth decay, gum abscesses if we didn’t include it. I just think that not everyone is very well educated in that department and caring for it. So I think, as a health professional, that we are promoting good health for them and for any future generations of that family.’ (GP1) | Providers express concern over the potential negative health outcomes for women if oral health is not addressed, indicating a recognition of oral health as an important aspect of ANC. |
| **Automatic motivation** | Emotion | **Provider level:**  Sense of professional satisfaction and purpose | ‘Yes, it would make me feel good. If I can do things in a better way, be more thorough and help people have a better pregnancy. We always want to be a better clinician at the end of the day.’ (OB1) | A sense of professional fulfillment motivates providers to include oral health discussions in ANC. They feel a sense of purpose when contributing to comprehensive patient well-being which enhances their commitment to integrating oral health in ANC. |
|  |  |  | ‘It is good when we all work together to get a woman through her pregnancy. It’s why we do what we do, isn’t it?’ (MW9) |  |
|  |  |  | ‘I think in any health care, it’s about being opportunistic, looking for opportunities to help a woman improve her health care in every way. And so taking a history for pregnancy is a great opportunity to identify other areas that she may not have been caring for or may have some vulnerabilities in. We could be really valuable to their pregnancy journey and getting them on the right track.’ (MW3) |  |

Abbreviations: ANC=Antenatal care; CPD=Continuing professional development; GP=General Practitioner.
